# CatLet Score as a Predictor of Cardiac Death in Patients With Acute Myocardial Infarction: Insights From Interpretable Machine Learning Models

**DOI:** 10.31083/RCM43310

**Published:** 2025-12-16

**Authors:** Xing-Hong Lin, Xue-Cheng Song, Xin Xu, Ruo-Nan Xu, Cai-Yun Song, Yong-Ming He

**Affiliations:** ^1^Department of Cardiology, The First Affiliated Hospital of Soochow University, 215000 Suzhou, Jiangsu, China; ^2^Department of Cardiology, The Third People’s Hospital of Bengbu, 230061 Bengbu, Anhui, China; ^3^Department of Cardiology, Shenzhen Nanshan District Peoples Hospital, 518000 Shenzhen, Guangdong, China; ^4^Department of Cardiology, The Hospital of Sihong, 223900 Suqian, Jiangsu, China

**Keywords:** catlet score, cardiac death, machine learning, prediction model

## Abstract

**Background:**

Predicting cardiac death in patients with acute myocardial infarction (AMI) remains a major challenge. The Coronary Artery Tree description and lesion evaluation (CatLet) angiographic scoring system can describe the variability in coronary artery anatomy, the degree of stenosis of the affected coronary artery, and the subtended myocardial territory. Therefore, this study aimed to establish an effective and interpretable machine learning (ML) model to explore the relationship between the CatLet score and cardiac death in patients with AMI.

**Methods:**

The CatLet score was calculated in 767 consecutively enrolled patients with AMI. Cox regression analysis, Kaplan–Meier survival analysis, and restricted cubic spline analysis were used to explore the association between the CatLet score and cardiac death in patients with AMI. Six ML methods were used to build predictive models. The Shapley Additive Explanations (SHAP) analysis was used to visualize model features and individual case predictions.

**Results:**

Compared to the lowest CatLet score of tertile 1, patients with the highest CatLet score (tertile 3) had a higher risk of cardiac death (hazard ratio (HR) = 3.71; 95% confidence interval (CI) = 1.36–10.08; *p* = 0.010). Restricted cubic spline analysis indicated a linear association between the CatLet score and cardiac death. The ML results showed that the adaptive boosting (Adaboost) model had the most reliable performance with an area under the curve (AUC) of 0.927, a sensitivity of 0.902, and a specificity of 0.796. The SHAP analysis showed that the CatLet score was a significant contributor to the cardiac death outcome.

**Conclusions:**

The Catlet score positively correlates with the risk of cardiac death in patients with AMI, while the use of ML modeling can effectively predict the risk of cardiac death.

## 1. Introduction 

Acute myocardial infarction (AMI) is one of the leading causes of cardiovascular death worldwide [1]. Despite significant advances in diagnostic and therapeutic techniques, cardiac mortality in AMI patients remains high [2, 3]. Therefore, identification of patients at risk of cardiac death remains a major challenge [4]. AMI is mainly caused by coronary blood flow limitation, which has been considered the main determinant of patient survival [5]. Previous studies have shown that the Synergy between Percutaneous Coronary Intervention with Taxus and Cardiac Surgery (SYNTAX) score is a risk stratification tool based on the anatomical features of coronary artery lesions, which can assess the complexity and severity of coronary artery lesions [6, 7]. However, the SYNTAX score, based solely on the left or right dominance, did not adequately describe the variability of coronary anatomy, much less evaluate the extent of coronary blood supply [8, 9, 10]. Recently, a new Coronary Artery Tree description and Lesion Evaluation (CatLet) angiographic scoring system has been developed, which is unique in that the importance of a coronary is weighted according to the number of myocardial segments it perfuses so as to account for and semi-quantify the coronary variation [11]. Previous studies have shown that the CatLet score is useful in explaining the variability of coronary artery anatomy, assessing the severity and complexity of the diseased coronary tree, and predicting long-term outcome in AMI patients with satisfactory reproducibility [11].

In recent years, machine learning (ML) has been introduced into medical predictions, and various disease prediction models have been developed with good prediction performance [12, 13, 14]. These studies showed that the combination of ML and clinical data could provide a better understanding and build useful prediction models to support decision-making [15, 16]. However, the potential of ML techniques in evaluating the relationship between the CatLet score and cardiac death in AMI patients has not been fully explored. Therefore, an ML prediction model was developed for cardiac death in AMI patients based on the CatLet score in this study. Shapley Additive Explanations (SHAP) analysis is a new method for interpreting ML models based on game theory, which is capable of both local and global explanations and has been validated in other studies. Therefore, the current study combined ML models based on the SHAP analysis to determine the contribution of the CatLet score to the identification of cardiac death.

## 2. Materials and Methods

### 2.1 Study Design and Study Population

Consecutive patients with suspected acute myocardial infarction (ST-segment or non-ST-segment elevation), admitted to this hospital and who underwent coronary angiography, were retrospectively enrolled from January 1, 2012, to September 30, 2015. AMI was diagnosed according to the third universal definition of myocardial infarction [17]. Exclusion criteria included: (1) incomplete key clinical data; (2) history of coronary artery bypass grafting; (3) congenital heart disease and other heart diseases requiring surgical intervention; (4) poor CAG images; and (5) lost to follow-up. A total of 45 clinical variables and angiographic variables were collected from the electronic medical record system of the First Affiliated Hospital of Soochow University, according to the CatLet angiographic scoring system. Variables included patient demographics, medical and personal histories, clinical examination findings, coronary anatomy, and lesion-related adverse angiographic features.

### 2.2 The CatLet Angiographic Scoring System 

The CatLet angiographic scoring system has been described in full elsewhere. In brief, this comprehensive angiographic scoring system has been developed based on the model of 17 myocardial segments, the law of competitive supply, and the law of flow conservation. A total of 54 (6 × 3 × 3) types of coronary circulation are defined based on the CatLet angiographic scoring system. Specifically, six right coronary artery (RCA) types are classified as: posterior descending artery (PDA) zero, PDA only, small RCA, average RCA, large RCA, and super RCA. Three left anterior descending artery (LAD) types are classified as short LAD, average LAD, and long LAD. The three diagonal branches (Dx) classifications included small Dx, inter. Dx, and large Dx. The coronary artery segments are weighted by the number of myocardial segments they perfuse to explain and semi-quantify coronary variation. The weights of the coronary segments are obtained using an online calculator or by mental calculation. The lesion score is the product of the weighting factor of the coronary segment and its degree of stenosis (2.0 for nonocclusive lesions and 5.0 for occlusive lesions). The scores of individual lesions are then summed to give a total score. In addition to scoring the lesions, the system also qualitatively records those adverse angiographic characteristics of the lesions, such as severe calcification, culprit vessels, severe distortion, angulation, bifurcation lesions, trifurcation, and thrombus burden. The CatLet score calculator is available at http://www.catletscore.com.

### 2.3 Outcomes and Study Variables

The primary outcome was cardiac death, defined as death due to AMI, heart failure, arrhythmia, other cardiovascular causes, or unexpected sudden death without an apparent noncardiac cause [18]. All patients were followed up for this outcome for 4 years. The clinical variables were age, sex, smoking and drinking status, body mass index (BMI), serum albumin, serum creatinine, left ventricular ejection fraction (LVEF), history of stroke, history of diabetes, and hypertension. Angiographic variables were heavy calcification and culprit vessels.

### 2.4 ML Model Building and Assessment

The dataset was divided into training and testing sets at a ratio of 7:3. The training dataset was used to build the model, and the testing dataset was used for validation. Initially, we applied the least absolute shrinkage and selection operator (LASSO) regression, a method that performed variable selection and coefficient shrinkage through regularization [19]. LASSO regression performed feature selection by reducing the coefficients of less significant features to zero, thereby effectively eliminating them from the model, and utilized 10-fold cross-validation to determine the best lambda value, thereby minimizing the average cross-validation error [20]. Subsequently, we implemented Boruta feature selection, a random forest-based algorithm that identified all relevant variables by comparing the importance of original features with randomly generated “shadow features” [21]. To ensure a robust and concise model, we chose the intersection of features identified by LASSO regression and the Boruta algorithm as our final set of predictor variables. We constructed and tested six ML algorithms, including k-nearest neighbors (KNN), support vector machine (SVM), adaptive boosting (Adaboost), light gradient boosting machine (LightGBM), extreme gradient boosting (XGBoost), and logistic regression (LR). The best performing model was selected to predict cardiac death in AMI patients. Model development utilized 10-fold cross-validation to improve reliability and generalization. The performance evaluation of the model included multiple indicators: area under the curve (AUC), accuracy, sensitivity, specificity, and F1 score. For all these performance measures, values range from 0 to 1, with higher scores indicating better model performance. After a thorough evaluation of model performance, we selected the model that showed the highest stability across all performance metrics as our final prediction model. To improve the interpretability of this model, we performed SHAP analysis, which provided insight into the importance and ranking of each variable [22]. SHAP values clearly showed the positive or negative impact of each variable on the model prediction with a screening threshold of 0.05. To address the issue of data imbalance, we adopted the random oversampling techniques and used the ROSE package in R software (version 4.4.3, The R Foundation for Statistical Computing, Vienna, Austria) to build new balanced training data.

### 2.5 Statistical Analysis

Participants were divided into three groups based on the tertiles of the CatLet score. Normally distributed continuous variables were expressed as mean ± standard deviation, while non-normally distributed continuous variables were expressed as the median and interquartile range (IQR). Categorical variables were expressed as counts (percentages). For the analysis of continuous variables, analysis of variance (ANOVA) was used for normally distributed data, while the Kruskal-Wallis test was applied for non-normally distributed data. Categorical variables were analyzed using the chi-square test. Multivariable Cox proportional hazards regression models were used to examine the association between CatLet score and cardiac death. Model 1 was not adjusted; Model 2 was adjusted for age and sex; Model 3 included all the covariates from Model 2 as well as primary hypertension, type 2 diabetes mellitus, BMI, prior stroke, smoking, drinking, serum albumin, serum creatinine, LVEF, and heavy calcification. Restricted cubic spline analysis was used to investigate the linear or nonlinear association between the CatLet score and cardiac death. All missing data were removed. Statistical analysis was performed using R software (version 4.4.3, The R Foundation for Statistical Computing, Vienna, Austria), and a two-tailed *p* < 0.05 was considered a statistically significant difference.

## 3. Results

### 3.1 Baseline Characteristics

**Fig. 1.**
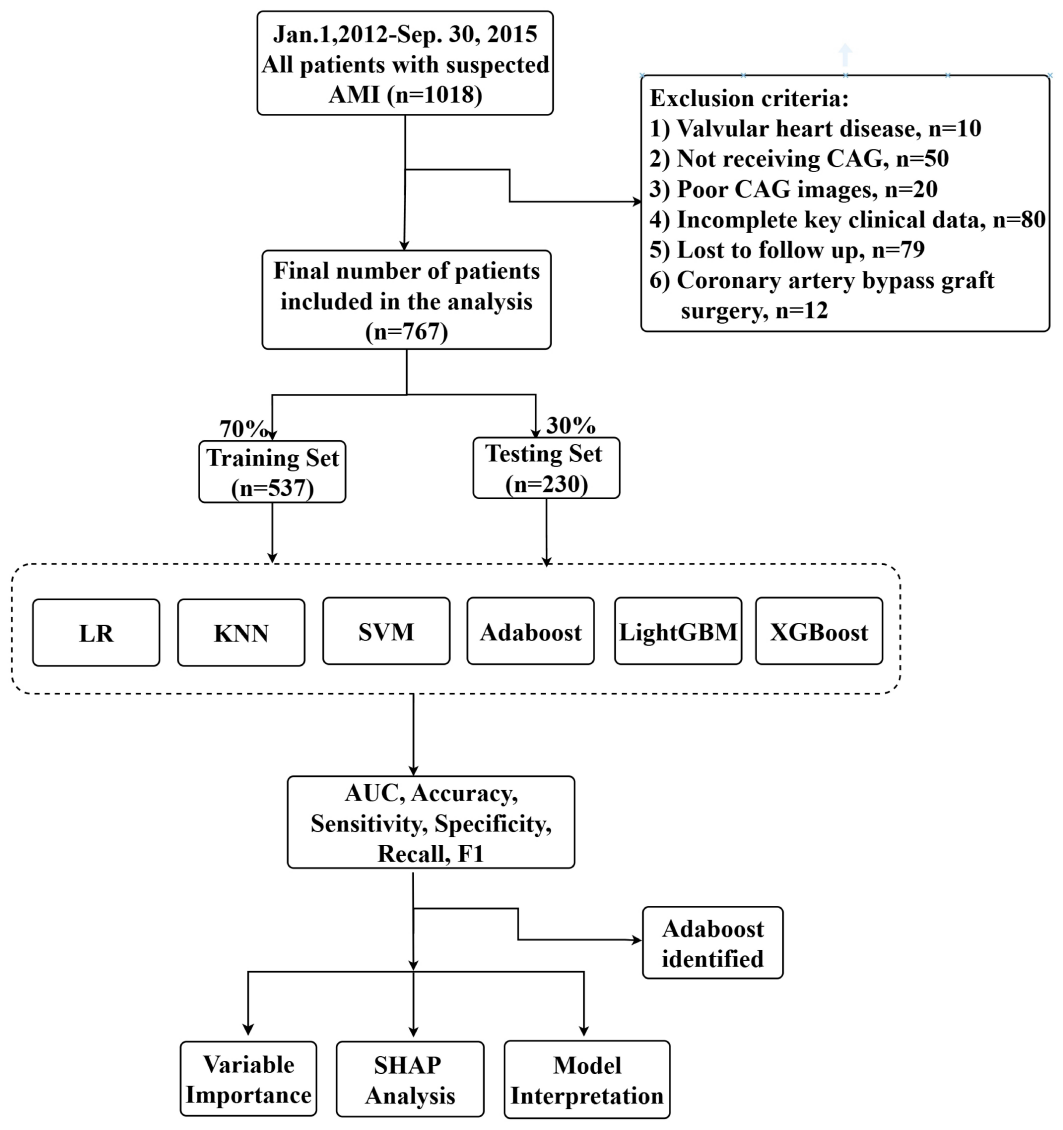
**The whole study workflow**. Abbreviations: KNN, k-nearest neighbors; SVM, support vector machine; Adaboost, adaptive boosting; LightGBM, light gradient boosting machine; XGBoost, extreme gradient boosting; LR, logistic regression; SHAP, Shapley Additive Explanations.

A total of 1018 patients were identified for potential analysis, of which 386 patients were excluded, and 767 patients finally met the inclusion criteria, as shown in Fig. 1. Table 1 shows the baseline characteristics stratified by the tertiles of CatLet score. The average age was 63.54 years. Notably, individuals with higher CatLet scores tended to be older, predominantly male, and had lower serum albumin levels, ejection fraction, and BMI. Furthermore, they exhibited a higher prevalence of hypertension, stroke, or heavy calcification of the lesion.

**Table 1.** **Characteristics of the study population based on the tertiles of CatLet score**.

Variables		Total	Q1 (≤12)	Q2 (13–18)	Q3 (≥19)	*p* value
		N = 767	299	232	236	
Age (y)		63.54 (12.25)	61.65 (12.52)	62.46 (12.80)	66.99 (10.57)	<0.001
Sex (n, %)						0.351
	Male	616.00 (80.31%)	247.00 (82.61%)	180.00 (77.59%)	189.00 (80.08%)	
	Female	151.00 (19.69%)	52.00 (17.39%)	52.00 (22.41%)	47.00 (19.92%)	
Dx (n, %)						0.123
	Inter.	536.00 (69.88%)	207.00 (69.23%)	173.00 (74.57%)	156.00 (66.10%)	
	Large	158.00 (20.60%)	57.00 (19.06%)	42.00 (18.10%)	59.00 (25.00%)	
	Small	73.00 (9.52%)	35.00 (11.71%)	17.00 (7.33%)	21.00 (8.90%)	
LAD (n, %)						0.315
	Average	443.00 (57.76%)	161.00 (53.85%)	137.00 (59.05%)	145.00 (61.44%)	
	Long	228.00 (29.73%)	93.00 (31.10%)	67.00 (28.88%)	68.00 (28.81%)	
	Short	96.00 (12.52%)	45.00 (15.05%)	28.00 (12.07%)	23.00 (9.75%)	
Dominance (n, %)						<0.001
	Average RCA	266.00 (34.68%)	126.00 (42.14%)	59.00 (25.43%)	81.00 (34.32%)	
	Large RCA	189.00 (24.64%)	51.00 (17.06%)	73.00 (31.47%)	65.00 (27.54%)	
	Small RCA	185.00 (24.12%)	86.00 (28.76%)	49.00 (21.12%)	50.00 (21.19%)	
	PDA only	45.00 (5.87%)	14.00 (4.68%)	17.00 (7.33%)	14.00 (5.93%)	
	PDA zero	44.00 (5.74%)	12.00 (4.01%)	16.00 (6.90%)	16.00 (6.78%)	
	Super RCA	38.00 (4.95%)	10.00 (3.34%)	18.00 (7.76%)	10.00 (4.24%)	
Drinking (n, %)						0.018
	Never	570.00 (74.32%)	207.00 (69.23%)	173.00 (74.57%)	190.00 (80.51%)	
	Past	25.00 (3.26%)	8.00 (2.68%)	8.00 (2.68%)	9.00 (3.81%)	
	Current	172.00 (22.43%)	84.00 (28.09%)	51.00 (21.98%)	37.00 (15.68%)	
Smoking (n, %)						0.209
	Never	277.00 (36.11%)	100.00 (33.44%)	90.00 (38.79%)	87.00 (36.86%)	
	Past	64.00 (8.34%)	19.00 (6.35%)	20.00 (8.62%)	25.00 (10.59%)	
	Current	426.00 (55.54%)	180.00 (60.20%)	122.00 (52.59%)	124.00 (52.54%)	
Heavy calcification (n, %)		81.00 (10.56%)	11.00 (3.68%)	12.00 (5.17%)	58.00 (24.58%)	<0.001
Stelev (n,%)						0.064
	non-STEMI	382 (49.80%)	154 (52%)	101 (44%)	127 (54%)	
	STEMI	385 (50.20%)	145 (48%)	131 (56%)	109 (46%)	
Culprit LM (n, %)		21 (9%)	0 (0%)	0 (0%)	21 (9%)	<0.001
Culprit LAD (n, %)		422.00 (55.02%)	162.00 (54.18%)	140.00 (60.34%)	120.00 (50.85%)	0.111
Culprit LCX (n, %)		150.00 (19.56%)	66.00 (22.07%)	33.00 (14.22%)	51.00 (21.61%)	0.049
Culprit RCA (n, %)		217.00 (28.29%)	73.00 (24.41%)	64.00 (27.59%)	80.00 (33.90%)	0.052
LAD (n, %)		423.00 (55.15%)	155.00 (51.84%)	142.00 (61.21%)	126.00 (53.39%)	0.080
LCX (n, %)		150.00 (19.56%)	62.00 (20.74%)	31.00 (13.36%)	57.00 (24.15%)	0.011
RCA (n, %)		252.00 (32.86%)	74.00 (24.75%)	71.00 (30.60%)	107.00 (45.34%)	<0.001
Primary Hypertension (n, %)		497.00 (64.80%)	181.00 (60.54%)	145.00 (62.50%)	171.00 (72.46%)	0.011
Prior stroke (n, %)		32.00 (4.17%)	8.00 (2.68%)	11.00 (4.74%)	13.00 (5.51%)	0.233
Type 2 diabetes mellitus (n, %)		171.00 (22.29%)	50.00 (16.72%)	60.00 (25.86%)	61.00 (25.85%)	0.012
BMI (kg/m^2^)		24.19 (3.64)	24.62 (3.90)	24.10 (3.67)	23.74 (3.21)	0.015
LDL-C (mmol/L)		2.59 (0.87)	2.51 (0.77)	2.63 (0.84)	2.66 (1.00)	0.103
Albumin (g/L)		38.49 (4.59)	39.15 (4.30)	38.56 (4.20)	37.58 (5.15)	0.002
Creatinine (umol/L)		75.72 (40.69)	77.26 (52.46)	72.40 (22.41)	77.01 (37.39)	0.147
LVEF (%)		54.12 (11.40)	55.94 (10.98)	53.73 (11.51)	52.19 (11.51)	<0.001

Abbreviations: CatLet, Coronary Artery Tree description and Lesion Evaluation and Treatment System; BMI, body mass index; LDL-C, low density lipoprotein-cholesterol; Dx, diagonal branches; LAD, left anterior descending artery; RCA, right coronary artery; LVEF, left ventricular ejection fraction; LCX, left circumflex; PDA, posterior descending artery. Q1, Q2, and Q3, Tertile partitioning of the CatLet score.

### 3.2 Associations Between the CatLet Score and Cardiac Death

Multivariable Cox proportional hazards models were used to assess the impact of CatLet score on cardiac death after adjusting for all covariates (model 3). CatLet score was significantly associated with the risk of cardiac death (hazard ratio (HR): 1.05, 95% CI: 1.02–1.09, *p* = 0.002, per 1 point increase). On the categorical scale, as compared with Q1, the HRs for cardiac death were 2.06 (0.68–6.23) for Q2 and 3.71 (1.36–10.08) for Q3 (*p* for trend = 0.004), respectively (Table 2).

**Table 2.** **Multivariate Cox regression analysis of CatLet score with cardiac death**.

Characteristic		Model 1		Model 2		Model 3	
		HR (95% CI)	*p* value	HR (95% CI)	*p* value	HR (95% CI)	*p* value
Continuous							
	CatLet score	1.09 (1.05–1.12)	<0.001	1.07 (1.03–1.10)	<0.001	1.05 (1.02–1.09)	0.002
	(per1point increase)						
Categories							
	Q1	1 (Ref)		1 (Ref)		1 (Ref)	
	Q2	1.76 (0.69–4.44)	0.232	1.59 (0.62–4.05)	0.333	2.06 (0.68–6.23)	0.201
	Q3	4.97 (2.20–11.27)	<0.001	3.77 (1.67–8.50)	0.001	3.71 (1.36–10.08)	0.010
*p* for trend			<0.001		<0.001		0.004

The Model 1 was unadjusted. The Model 2 was adjusted for age, sex. The Model 3 was further adjusted for covariates in Model 2, plus primary hypertension, type 2 diabetes mellitus, BMI, prior stroke, smoking, drinking, serum albumin, serum creatinine, LVEF, and heavy calcification. Abbreviations: CatLet, Coronary Artery Tree description and Lesion Evaluation and Treatment System; BMI, Body mass index; HRs, hazard ratios; LVEF, left ventricular ejection fraction; CI, confidence interval.

### 3.3 Analysis of Nonlinear Relationship

**Fig. 2.**
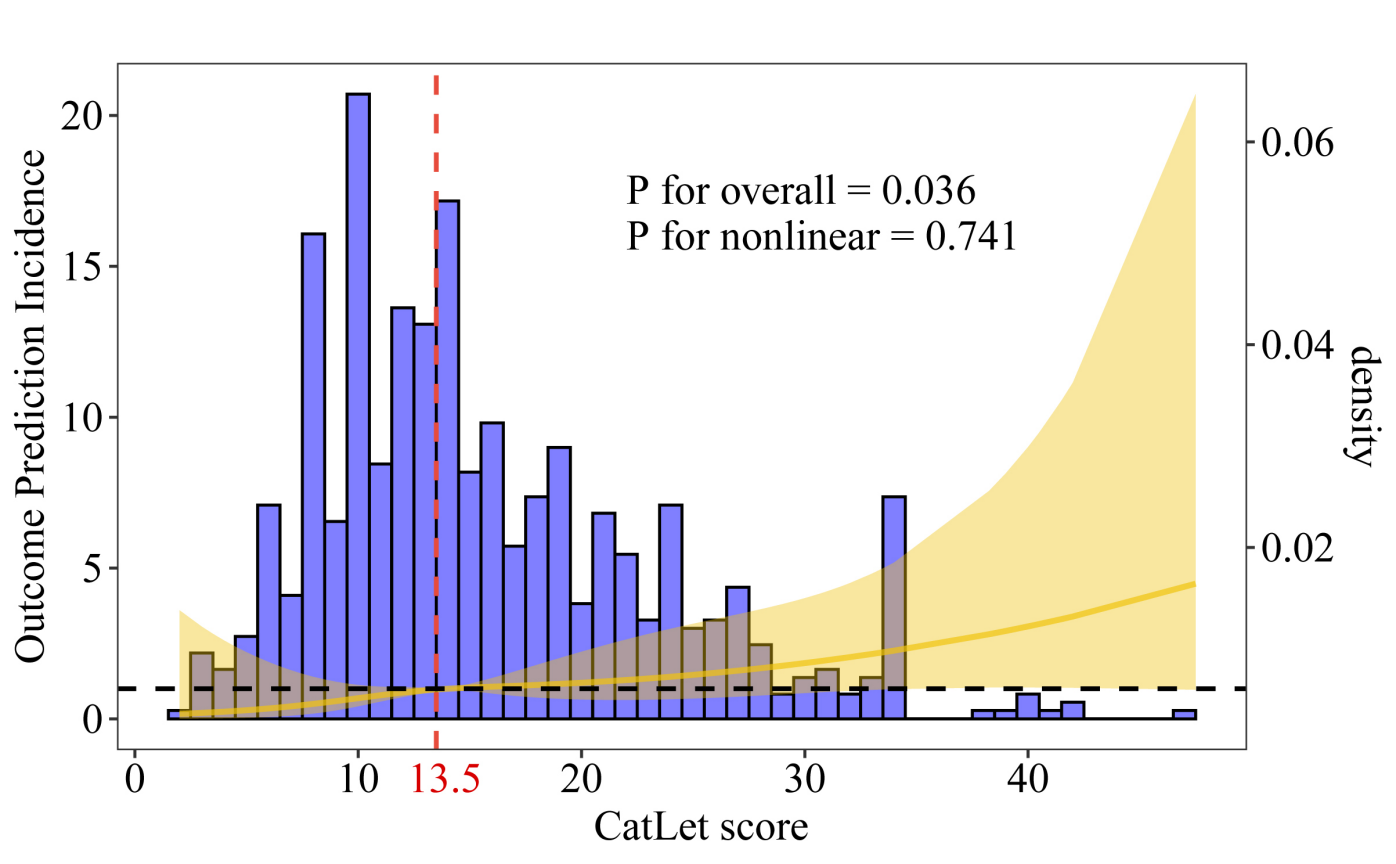
**Distribution plot of the CatLet score and the relationship between the CatLet score and cardiac death using a restricted cubic spline regression model**.

**Fig. 3.**
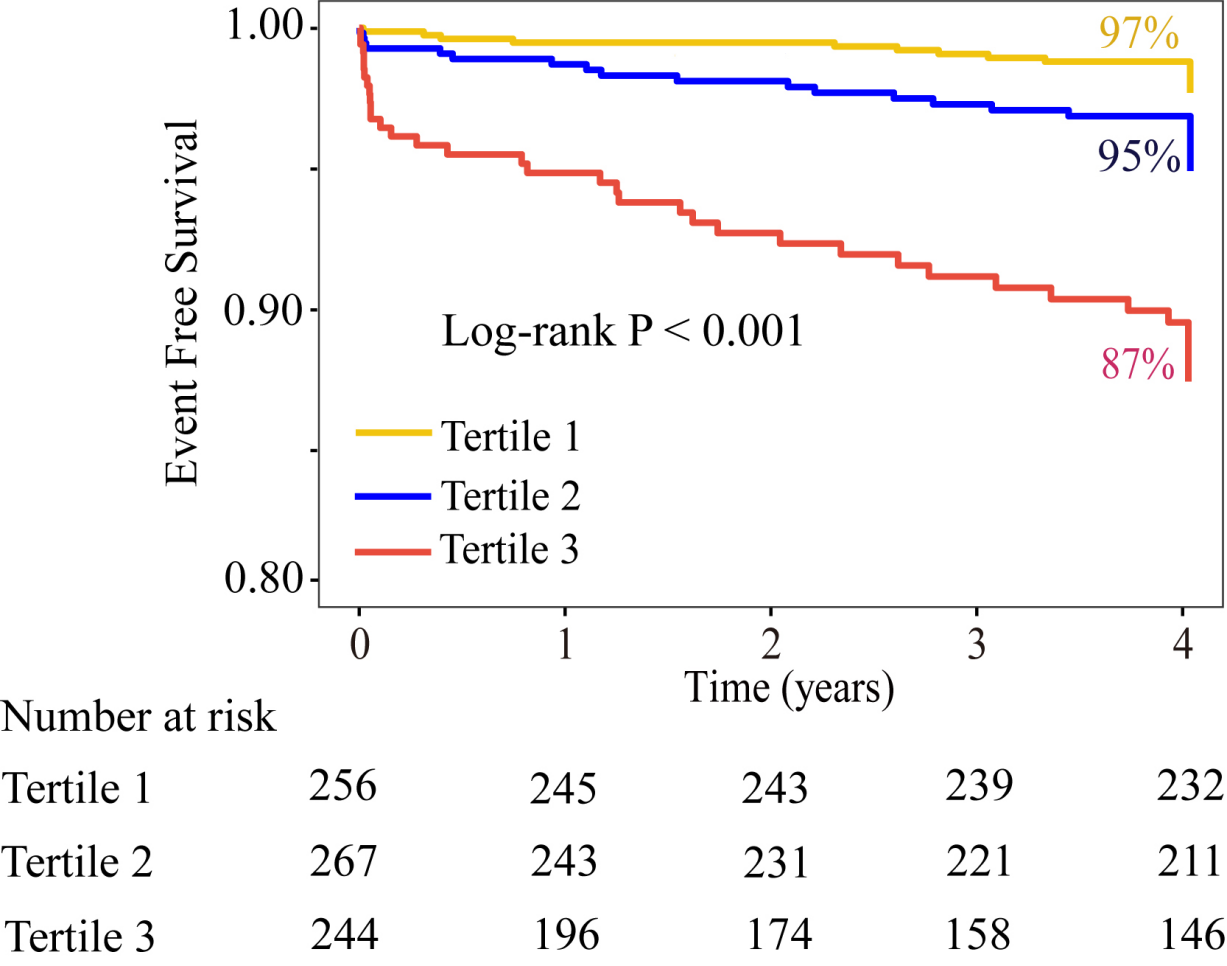
**Kaplan-Meier survival curves for cardiac death stratified by the tertiles of CatLet score**.

Using the restricted cubic spline analysis, the linear trend between the CatLet score and cardiac death was revealed (*p* for overall = 0.036; *p* for nonlinear = 0.741) (Fig. 2). The threshold effect analysis determined the critical level of the CatLet score to be 13.5. The Kaplan-Meier survival curves showed that the survival probability decreased with the increase of the tertiles of CatLet score (*p* < 0.001) (Fig. 3).

### 3.4 Feature Selection

**Fig. 4.**
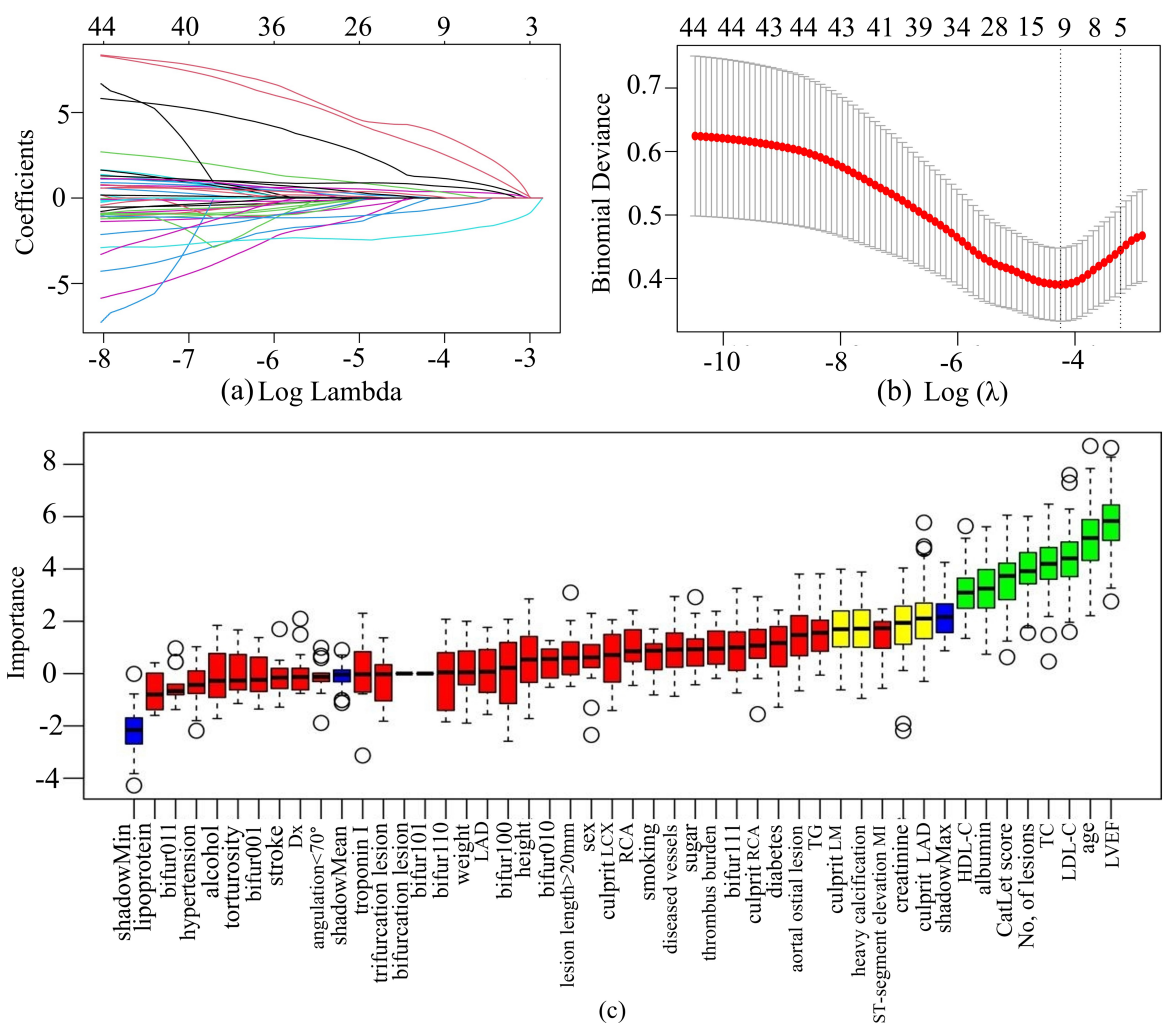
**Feature selection by LASSO regression and Boruta’s algorithm**. (a) The variation characteristics of the LASSO coefficient. The different colored lines represented the different variables. (b) Optimization parameters (λ) of the LASSO model were selected by 10-fold cross-validation. The left dashed line represented λ min (minimum cross-validated error), while the right dashed line indicated λ1se (the largest λ within one standard error of λ min). (c) Feature identification via the Boruta algorithm. The X-axis represented all features, and the Y-axis was the Z-value of each feature. The green boxes represented significant variables, while the yellow ones denoted tentative, and the red ones indicated unimportant. The circles represent the fluctuation of feature importance in multiple random iterations, indicating exceptionally high or low individual importance score.

LASSO regression was used to select features in the training set, and the characteristics of variable coefficients were shown in Fig. 4a. As shown in Fig. 4b, the iterative analysis was carried out by a ten-fold cross-validation method. The LASSO regression identified 11 variables. The Boruta algorithm recognized 12 variables, as shown in the green and yellow boxes in Fig. 4c. By intersecting the variables derived from the two algorithms, a total of 7 variables were finally used to build the ML model. These variables included serum creatinine, LVEF, age, serum albumin, CatLet score, heavy calcification, and culprit LAD.

### 3.5 Machine Model Development

**Fig. 5.**
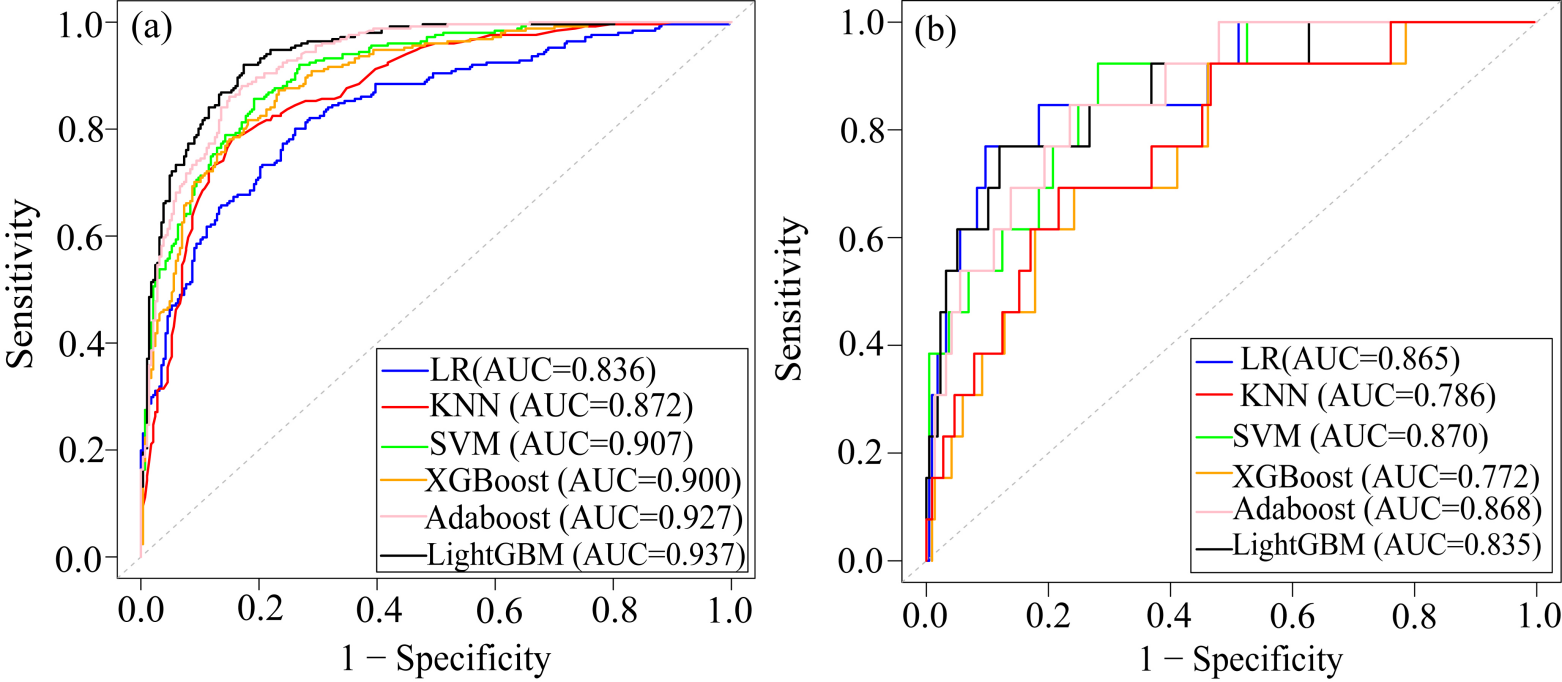
**ROC of the six ML models**. (a) ROC of the training dataset. (b) ROC of the test dataset. Abbreviations: ROC, receiver operating characteristic; AUC, area under the curve; KNN, k-nearest neighbors; SVM, support vector machine; Adaboost, adaptive boosting; LightGBM, light gradient boosting machine; XGBoost, extreme gradient boosting; LR, logistic regression.

As shown in Table 3 and Fig. 5, KNN, SVM, and LightGBM models all performed well on the training set, but their performances declined on the test set. The XGBoost model demonstrated superior performance on the training set, with an AUC of 0.900, accuracy of 0.903, sensitivity of 0.941, specificity of 0.860, and F1 score of 0.912. However, its performance on the test set showed a significant drop, particularly in AUC (0.772), indicating a potential over-fitting. Adaboost had the highest F1 score (0.916), suggesting that it achieved the best balance between precision and recall on the test set. The AUC, accuracy, and sensitivity of the Adaboost model were higher than those of LR in the test set. Therefore, the Adaboost model was selected as the best model to predict cardiac death in AMI patients. The results of the statistical comparison of ROC curves of each model were shown in the **Supplementary Tables 1**,**2**.

**Table 3.** **Evaluation of the performance of the six ML models**.

Module	Data set	AUC	Accuracy	Sensitivity	Specificity	PPV	F1 score
LR	train	0.836	0.767	0.850	0.672	0.742	0.796
	test	0.865	0.839	0.843	0.769	0.766	0.908
KNN	train	0.872	0.773	0.829	0.708	0.769	0.796
	test	0.786	0.826	0.848	0.462	0.780	0.902
SVM	train	0.907	0.827	0.861	0.788	0.870	0.842
	test	0.870	0.843	0.857	0.615	0.850	0.912
LightGBM	train	0.937	0.844	0.871	0.812	0.895	0.829
	test	0.835	0.822	0.834	0.615	0.850	0.781
XGBoost	train	0.900	0.903	0.941	0.860	0.918	0.912
	test	0.772	0.843	0.853	0.692	0.900	0.911
Adaboost	train	0.927	0.853	0.902	0.796	0.900	0.868
	test	0.868	0.852	0.862	0.692	0.912	0.916

ML, machine learning; LR, logistic regression; KNN, k-nearest neighbors; SVM, support vector machine; LightGBM, light gradient boosting machine; XGBoost, extreme gradient boosting; AUC, area under the curve; PPV, positive prediction value.

### 3.6 Model Interpretation

**Fig. 6.**
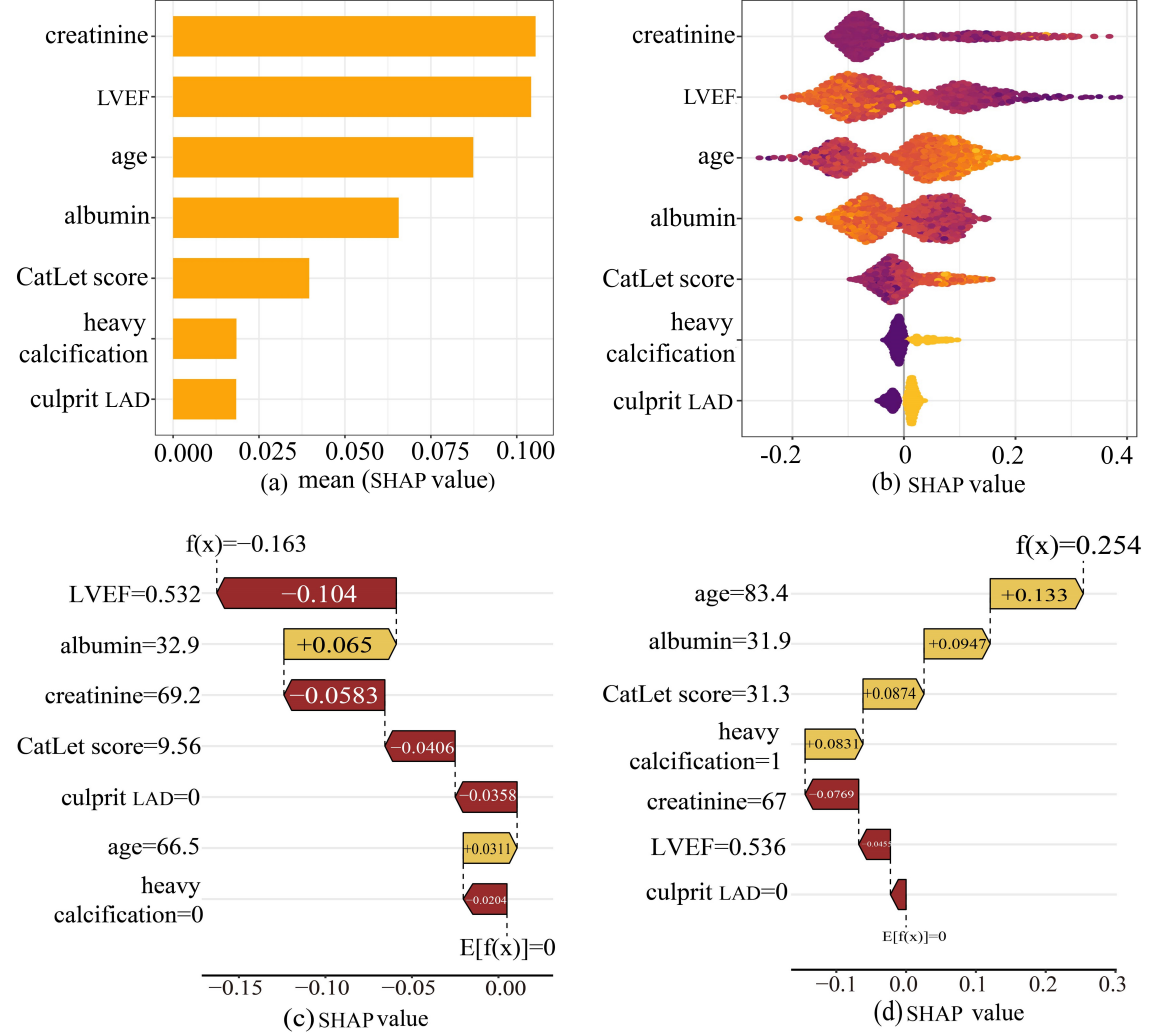
**The SHAP analysis of the Adaboost model**. (a) A bar plot displaying the mean SHAP value for variables. (b) The beeswarm plots displayed the distribution of variables. (c) SHAP waterfall plot for case 1. (d) SHAP waterfall plot for case 2. Abbreviations: SHAP, Shapley Additive Explanations, and Adaboost, adaptive boosting.

We conducted an interpretable analysis of the Adaboost model. The importance of each feature was ranked from highest to lowest according to the SHAP analysis (Fig. 6a). SHAP values represented the influence of each feature on the final prediction result. Fig. 6b showed the influence of each feature on the model output. Each dot in a row symbolized a patient, and its color denoted the feature value: yellow for larger values and purple for lower values. The more scattered the points of the graph, the greater the influence of the variables on the model. In our study, older age, higher CatLet scores, culprit LAD, and heavy calcification were associated with increased risk of cardiac death, while elevated levels of albumin and LVEF served as protective factors.

To elucidate the decision-making process at the individual level, SHAP waterfall plots were utilized to conduct local interpretable analysis on two representative cases randomly selected from the test set. For one case (Fig. 6c), the model significantly reduced the mortality risk for a 66-year-old patient (from baseline f(x) = 0 to final prediction f(x) = –0.163). The most significant protective factor was non-culprit LAD (0, SHAP value: –0.0358), a lower CatLet score (9.56, SHAP value: –0.0406), and no heavy calcification (0, SHAP value: –0.0204), indicating relatively mild disease severity. Creatinine level (69.2 umol/L, SHAP value: –0.0583) and ejection fraction remained stable (0.532, SHAP value: –0.104). The adverse factor was slightly below-normal levels of albumin (32.9 U/L, SHAP value: +0.065). In contrast, case 2 (Fig. 6d) presented an 83-year-old patient, where the model indicated an increased risk of death (compared to the baseline E[f(x)] = 0, the final predicted f(x) = 0.254). Significant risk factors included older age (83, SHAP value: +0.133), higher CatLet score (31.3, SHAP value: +0.0874), and severe calcification (1, SHAP value: +0.0831). The SHAP value quantified the contribution of each feature, with positive values (yellow bars) indicating risk factors and negative values (magenta bars) indicating protective effects.

## 4. Discussion

Key findings of the current study were as follows: (1) CatLet score was significantly associated with cardiac death, even after adjusting for covariates; (2) A linear relationship between the CatLet score and cardiac death was revealed with a cut-off value of 13.5; (3) Adaboost was the best machine learning model for predicting cardiac death in AMI patients compared with other machine learning models; (4) SHAP analysis showed that the combination of higher CatLet score, older age, heavy calcification of the lesion and the culprit vessel of LAD showed a synergistic effect on cardiac death. (5) The SHAP plot assigned a contribution value to each feature, indicating its relative importance to cardiac death in the current study, ranked as follows: serum creatinine, LVEF, age, serum albumin, CatLet score, heavy calcification, and culprit LAD.

In the current study, a higher CatLet score was associated with a higher incidence of cardiac death (HR = 3.71, *p* = 0.01), which was consistent with the results of previous studies of the Catlet score (HR = 3.95, *p* < 0.01) [23]. The identification of the CatLet score at 13.5 as the critical threshold was clinically important, and once this threshold was exceeded, the risk of death was significantly elevated. For high-risk patients with a CatLet score >13.5, aggressive intervention may be required. For low-risk patients with a CatLet score ≤13.5, overtreatment can be avoided, allowing for specific resource allocations.

We used ML models in combination with interpretable SHAP charts to explore the potential predictive power of the CatLet score for cardiac death. Of the six ML models considered, the Adaboost model achieved the best performance as it identified a combination of predictors reflecting cardiac death. The CatLet score has been found to have comparable predictive power and better calibration compared with the SYNTAX score in previous studies, leading to significant improvement in risk stratification of patients with AMI [11]. In addition, our study identified the culprit vessel of LAD as a major risk factor for cardiac death. Diseased LAD has long been considered one of the leading predictors in patients with CAD. According to the CatLet angiographic scoring system, short LAD subtended at least five myocardial segments relative to the 17 myocardial segments, still accounting for 30% of total left ventricle mass even in patients with a dominant RCA or a robust collateral circulation [23, 24]. Patients with LAD infarction had a higher 1-year mortality than those with RCA or LCX infarction, and were associated with an increased risk of heart failure and stroke [25]. Unsurprisingly, LAD involvement is also revealed to be an important predictor in the current study. In addition, Coronary artery calcification, Serum albumin, serum creatinine, LVEF, and age were consistent with the previous literature [26, 27, 28, 29]. The Adaboost model was constructed by these 7 variables, and the results showed that both the training set (AUC = 0.927) and the test set (AUC = 0.868) performed well, while the AUC of age, creatinine, ejection fraction, and CatLet score in the previous models for predicting cardiac death in AMI patients was 0.829 [23].

Compared with previous risk models, the machine learning model based on the CatLet score considered the intricate interactions between variables and can be dynamically adjusted according to individual patient characteristics. No prediction models adequately considering both coronary anatomy variability and its diseased severity and complexity have been reported. Therefore, an online computing tool can be developed based on the CatLet score machine learning model to generate personalized cardiac death risk and alert high-risk cases to cardiologists and intensive care teams, which will benefit clinicians in identifying high-risk patients early so as to intervene in time and improve long-term survival outcomes. In addition, our case analysis also demonstrated the potential of this model for clinical application.

Our study had some limitations. First, the retrospective and observational nature of our study may have led to inevitable selection bias. This study did not consider some factors, such as lifestyle and socioeconomic status, which were significantly related to the risk of cardiovascular disease. Future research will be needed to further incorporate these factors into the model. Second, due to the relatively small sample size and limited number of events, there was a risk of over-fitting in the XGBoost model and LightGBM model. However, we adopted 10-fold cross-validation and adjusted model parameters to alleviate over-fitting. Third, we employed advanced machine learning technologies, including Adaboost, to develop sophisticated models with robust computational and fitting capabilities. However, the current model was trained and tested on the same dataset. To better validate its generalizability and robustness across different environments, we will conduct external validation using larger samples, prospective, and multicenter cohort studies in the future.

## 5. Conclusions

The Adaboost model showed good performance for cardiac death after PCI in AMI patients. Our findings suggested that serum creatinine, LVEF, age, serum albumin, CatLet score, heavy calcification, and culprit LAD were closely associated with cardiac death in AMI patients.

## Availability of Data and Materials

The datasets used and analyzed during the current study are available from the corresponding author on reasonable request.

## Supplementary Material

Supplementary material associated with this article can be found, in the online version, at https://doi.org/10.31083/RCM43310.
