# Supplementary material for: CatLet Score as a Predictor of Cardiac Death in Patients With Acute Myocardial Infarction: Insights From Interpretable Machine Learning Models
Source: Rev Cardiovasc Med. 2025 Dec 16;26(12):43310. doi: 10.31083/RCM43310 (PMC12781014; doi:10.31083/RCM43310)
Supplement: Supplementary file 1 [file 2153-8174-26-12-43310-s1.docx]

**Supplementary Table 1. *p*-values from for pairwise AUC comparisons of train dataset between machine learning models using the Delong method.**

| Models | LR | KNN | SVM | LightGBM | XGBoost | Adaboost |
| --- | --- | --- | --- | --- | --- | --- |
| LR | - |  |  |  |  |  |
| KNN | ** | - |  |  |  |  |
| SVM | *** | ** | - |  |  |  |
| LightGBM | *** | *** | ** | - |  |  |
| XGBoost | *** | ** | >0.05 | ** | - |  |
| Adaboost | *** | *** | ** | * | ** | - |

P < 0.05^*^, P < 0.01^**^, P < 0.001^***^.

**Supplementary Table 2. *p*-values from for pairwise AUC comparisons of test dataset between machine learning models using the Delong method.**

| Models | LR | KNN | SVM | LightGBM | XGBoost | Adaboost |
| --- | --- | --- | --- | --- | --- | --- |
| LR | - |  |  |  |  |  |
| KNN | *** | - |  |  |  |  |
| SVM | >0.05 | *** | - |  |  |  |
| LightGBM | ** | ** | ** | - |  |  |
| XGBoost | *** | * | *** | *** | - |  |
| Adaboost | >0.05 | *** | >0.05 | ** | *** | - |

P < 0.05^*^, P < 0.01^**^, P < 0.001^***^.
